# Successful removal of 3 aspirated teeth following emergency intubation in a critically ill patient: A rare case report

**DOI:** 10.1097/MD.0000000000043805

**Published:** 2025-08-22

**Authors:** Hamza A. Abdul-Hafez, Enas Samara, Lana Saed, Mahdi Kittaneh, Abdulkarim Adas

**Affiliations:** a Department of Medicine, Faculty of Medicine and Health Sciences, An-Najah National University, Nablus, Palestine; b Department of General Surgery, An-Najah National University Hospital, Nablus, Palestine; c Department of Cardiothoracic Surgery, An-Najah National University Hospital, Nablus, Palestine.

**Keywords:** case report, flexible bronchoscope, intensive care unit, teeth aspiration

## Abstract

**Rationale:**

Foreign body aspiration is a rare yet potentially life-threatening condition in critically ill patients. Aspiration of dental fragments, including teeth, is exceedingly uncommon but can lead to significant airway complications, requiring prompt recognition and tailored intervention. Reporting such cases contributes valuable insights into the challenges faced in critical care and highlights effective management strategies.

**Patient concern:**

A 72-year-old male was admitted to the intensive care unit with cardiogenic shock, multiorgan failure, and pneumonia. During emergency intubation, he aspirated 3 prosthetic teeth, posing a diagnostic and therapeutic challenge.

**Diagnosis:**

Chest radiography and a computed tomography scan revealed foreign bodies lodged in the right middle bronchus, including a dental implant embedded in the bronchial wall.

**Intervention:**

Initial extraction attempts using flexible bronchoscopy by the pulmonology team were unsuccessful. Subsequently, the thoracic surgery team performed bedside bronchoscopy under sedation via a tracheostomy. Using specialized graspers, 3 prosthetic teeth were successfully retrieved without complications. Post-procedure imaging confirmed complete removal.

**Outcome:**

The patient tolerated the procedure well, showing no immediate complications. His clinical condition steadily improved, and he was discharged to a rehabilitation center for further care.

**Lessons:**

This case highlights the importance of early recognition and multidisciplinary management of dental aspiration. A high index of suspicion is important in unexplained respiratory deterioration in intensive care unit patients. Flexible bronchoscopy remains the cornerstone for diagnosis and treatment, especially in critically ill individuals. Moreover, reporting rare events such as dental aspiration is important to enhance awareness among clinicians, refine diagnostic approaches, and inform tailored interventions. Multidisciplinary collaboration played an essential role in achieving a successful outcome in this critically ill patient.

## 1. Introduction

Foreign body aspiration into the tracheobronchial tree is a rare but potentially serious and life-threatening condition, especially in critically ill patients.^[1]^ Clinical features can range from asymptomatic to severe respiratory complications, even death. Among these, dental aspirations, including teeth or prosthetic fragments, are very rare, with significant risk due to their size, shape, and potential to obstruct the airway.^[2,3]^ This condition is more frequent in patients with maxillofacial trauma or introral procedures, in addition to posing a high risk in vulnerable populations such as the elderly, neurologically impaired, or mechanically ventilated patients.^[2–4]^

Here, we describe a rare case of a critically ill patient who was admitted to the intensive care unit (ICU) with shock, during which he aspirated 3 prosthetic teeth during oropharyngeal intubation. He underwent successful removal of the teeth from the right middle bronchus by flexible bronchoscopy. This unusual event posed diagnostic and therapeutic challenges in a complex patient and highlights the importance of a multidisciplinary team in managing such cases.

## 2. Case presentation

A 72-year-old male was admitted to the ICU after 12 days of hospitalization for deteriorated cardiogenic shock with multiorgan failure, including respiratory, renal, liver, and circulatory, secondary to non-ST elevation myocardial infarction and pneumonia. He was on vasopressors and intubated on mechanical ventilation during hospitalization.

On admission, the patient was hemodynamically unstable, presenting with an altered level of consciousness, severe respiratory distress, and hypoxemia. A diagnostic cardiac catheterization was performed, revealing significant coronary artery disease. Therefore, the patient underwent a complex rotablation to the left anterior descending artery and left circumflex artery. Following rotablation, the patient regained consciousness, demonstrated an intact cough reflex, and was successfully extubated. However, hours later, he developed sudden hypoxia and collapsed right lung, which failed to improve on a trial of noninvasive ventilation. As a result, an emergent intubation was performed due to respiratory deterioration. Attributed to generalized myopathy, failed extubation, and an anticipated prolonged weaning process, an uneventful tracheostomy was performed, with subsequent slow weaning from the ventilator.

**Figure 1.**
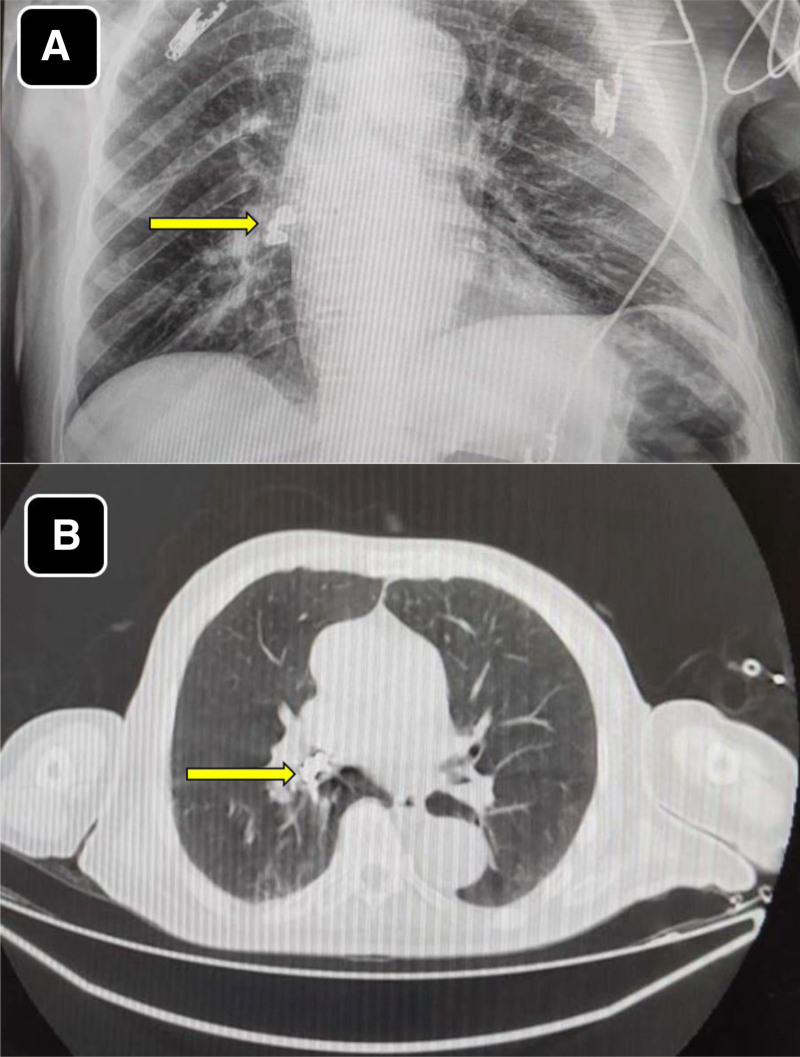
(A) Chest X-ray (AP view) showing the radiopaque tooth in the right-sided bronchus intermedius (yellow arrow). (B) Chest CT scan (lung view) showing the radiopaque tooth residing in the right-sided bronchus intermedius (yellow arrow).

Later, chest radiography revealed abnormal opacity in the right middle lung zone (Fig. 1A), prompting further investigation by non-contrast chest computed tomography (CT) scan. After he was stable, a chest CT scan was performed and showed a foreign body in the right middle bronchus, identified as a dental implant with an attached screw embedded in the bronchial wall, which mostly occurred during the second emergent intubation (Fig. 1B).

After ensuring the patient’s stability, initial attempts to extract the foreign body using flexible bronchoscopy by the pulmonology team were unsuccessful. Given the complex nature of the case and the patient’s critical condition, our thoracic surgery team was consulted. A decision was made to proceed with bedside bronchoscopy in the ICU, with readiness to perform video-assisted thoracoscopic surgery if required.

**Figure 2.**
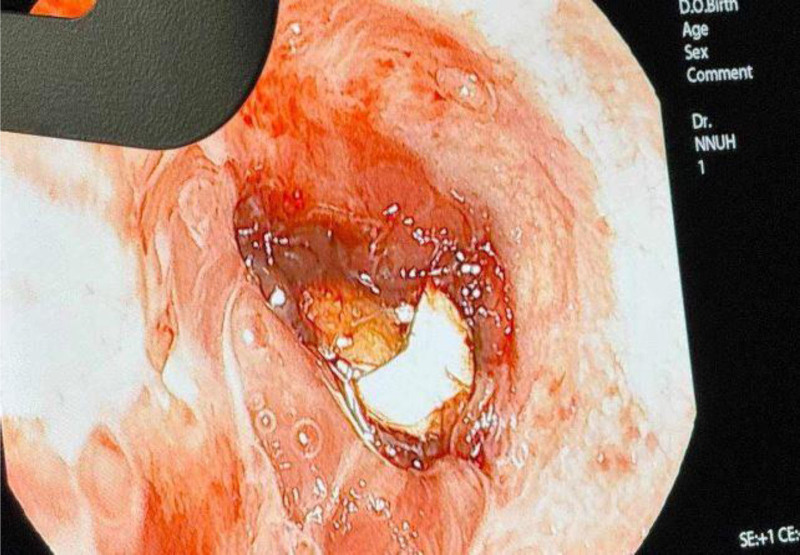
Bronchoscopic view of the tooth inside the right-sided bronchus intermedius before removal by flexible bronchoscopy.

**Figure 3.**
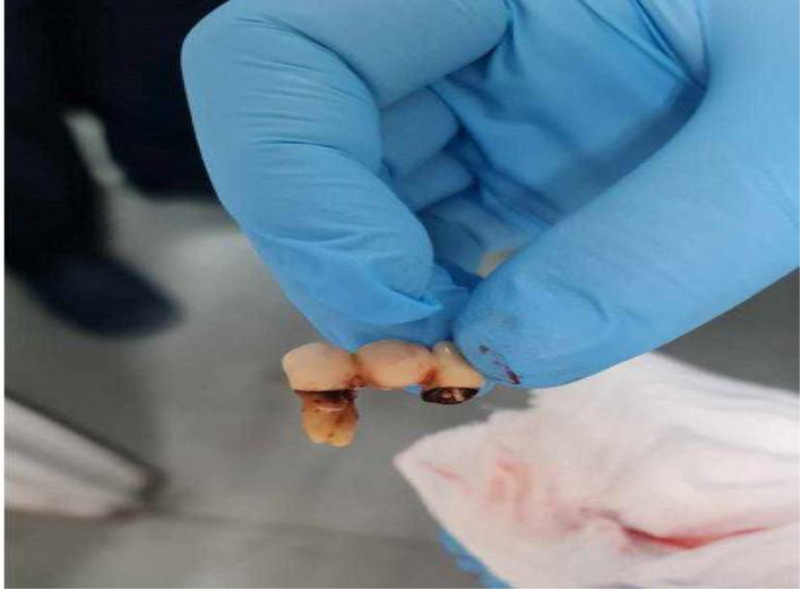
A real view of the tooth after removal from the right-sided bronchus intermedius.

Bedside flexible bronchoscopy was performed under sedation with the patient in the supine position, mechanically ventilated via an existing tracheostomy tube in the ICU. The tracheostomy tube served as the access port for the flexible bronchoscope. After saline irrigation and suctioning to clear secretions, direct visualization revealed 3 prosthetic teeth lodged in the right middle bronchus (Fig. 2, Video 1). Using a specialized grasper, we carefully retrieved the teeth, ensuring minimal trauma to the bronchial wall. After successfully removing all 3 teeth (Fig. 3), we replaced the tracheostomy tube with an 8F tube to provide ongoing respiratory support. The tracheostomy tube was changed during the procedure and again before transferring due to concerns of a potential cuff leak, ensuring an adequate seal for continued ventilation.

**Figure 4.**
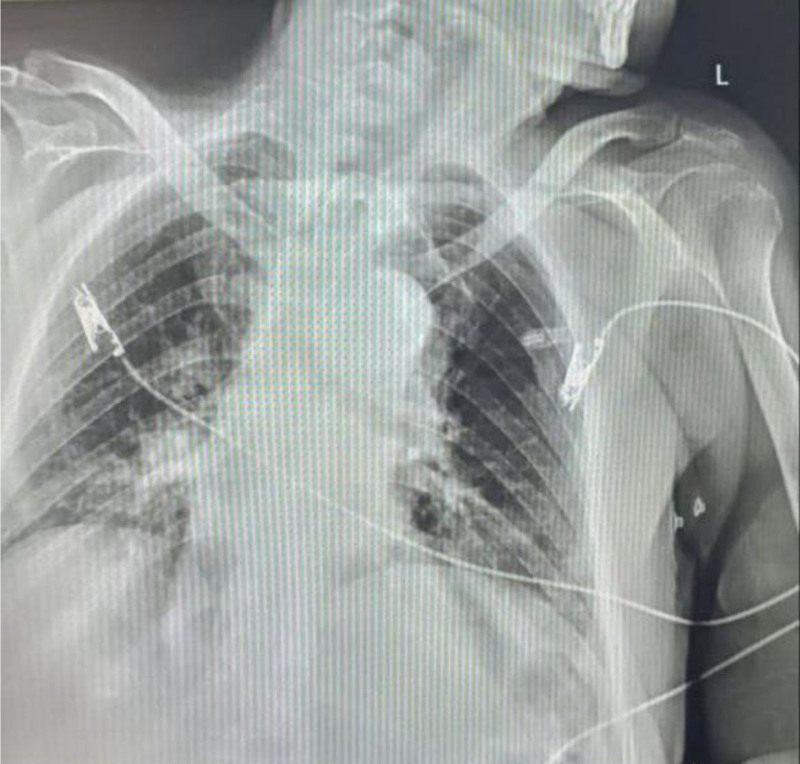
Post-procedural chest X-ray scan.

Post-procedure imaging, including chest X-ray, confirmed the absence of pneumothorax and complete removal of the foreign body (Fig. 4). A follow-up bronchoscopy was performed to evaluate the tracheobronchial tree for any residual debris or bleeding with additional saline irrigation and suctioning, which ensured a clear airway. The patient tolerated the procedure well without immediate complications and continued his ICU monitoring and management. Post-procedure, the patient received routine airway care including tracheostomy suctioning, nebulized bronchodilators (ipratropium, hexacarbon), and cold saline. He continued broad-spectrum antibiotics and antifungals per infectious disease guidance. Anticoagulation was managed cautiously due to airway bleeding risk. Heart failure therapy included bisoprolol, spironolactone, and furosemide. Renal function was monitored to prevent nephrotoxicity. Percutaneous endoscopic gastrostomy (PEG)-based nutrition was initiated and integrated with physiotherapy.

However, the presence of critical illness-related complications such as myopathy, gangrene, and a history of candidemia emphasizes the need for continued multidisciplinary care. The patient was in a relatively stable clinical and hemodynamic condition and was discharged 5 days post-procedure with a plan of transfer to a rehabilitation center for physical and occupational therapy, along with ongoing medical management.

Following discharge to rehabilitation, the patient underwent an initial assessment within 48 hours, followed by weekly multidisciplinary reviews for 1 month. Follow-up included bedside clinical evaluations, pulse oximetry, PEG site and tracheostomy care, and scheduled outpatient visits in cardiology, nephrology, and pulmonology. At 6 weeks post-procedure, the patient remained clinically stable and continued active rehabilitation.

## 3. Discussion

Emergency intubations have a higher incidence of complications such as aspiration, esophageal intubation, dental injury, and pneumothorax. For instance, emergency intubations have a 4.2% complication rate, with aspiration occurring in 2.8% of cases and dental injury in 0.2%.^[5]^ Tooth aspiration into the bronchial tree is a rare complication during intubation, especially in emergency settings where rapid sequence intubation may not allow pre-intubation assessment and preparation.^[6]^ It is more common in the elderly or those with predisposed characteristics such as maxillofacial trauma, loose teeth, or reduced airway reflexes.^[7]^ Prevention is the primary line of defense against foreign body aspiration. Proper dental evaluations before high-risk procedures, improved prosthetic design, and vigilant care during intubation can significantly reduce the risk.^[8]^

Several cases in the literature demonstrate clinical management and outcomes. Hadad et al reported a case of foreign body aspiration in an elderly patient during emergency orotracheal intubation. An 81-year-old man admitted for meningitis aspirated a dental prosthetic crown into the right lower lobe bronchus. The foreign body was discovered using chest radiography and removed with a flexible bronchoscope and basket clamp.^[8]^ Eliçora et al analyzed 15 cases of tracheobronchial tooth and dental prosthesis aspiration, with the majority (80%) removed using rigid bronchoscopy. The study underscored the importance of diagnostic bronchoscopy and intervention to prevent complications, especially in individuals with predisposed factors.^[4]^ Dhadge described a case of tooth aspiration during emergency endotracheal intubation of a 72-year-old male following cardiac arrest. The aspirated tooth was removed using a flexible bronchoscope from the right lower lobe bronchus.^[9]^

Critical care myopathy contributes significantly to prolonged ventilator dependence in critically ill patients.^[10]^ In this case, the patient’s critical care myopathy required a tracheostomy. While tracheostomy is often performed to allow long-term ventilator weaning by improving respiratory mechanics, reducing complications, and increasing patient comfort and mobility, all of which are important in managing patients with critical care myopathy,^[11]^ this patient’s myopathy was severe, resulting in swallowing difficulty and the necessity for enteral feeding using a PEG tube. The unique finding in this case was a metallic foreign body (dental prosthesis) discovered in the bronchus. Foreign body aspiration in intubated patients is rare and often underdiagnosed due to overlapping clinical features such as atelectasis and recurrent infections. In our patient, the foreign body caused right lower lobe atelectasis, which led to hypoxia and delayed ventilator weaning. The diagnosis was confirmed through chest CT, and therapeutic bronchoscopy was used to remove it successfully. Bronchoscopic removal is the gold standard for treating aspirated foreign materials, providing both diagnostic and therapeutic benefits. Flexible bronchoscopy is especially useful for peripheral airway foreign bodies or in unstable individuals, since it is minimally invasive and can be conducted under local anesthesia and sedation.^[12]^

Rigid bronchoscopy excels in therapeutic interventions, such as airway obstruction management and stent placement, due to its strong construction and larger channels, although it is confined to the central airways. Flexible bronchoscopy, which is commonly used for diagnostic procedures such as biopsies and lavage, is more adaptable, accessing peripheral airways under minimal sedation. Recent improvements have enhanced its therapeutic usage. Both modalities complement one another, with clinical demands, pathological location, and procedural goals guiding their selection.^[13]^ This case highlights the importance of maintaining a high index of suspicion in patients with unexplained respiratory deterioration, particularly those with tracheostomies or prolonged intubation.

Despite the complexity of this case, the patient’s condition steadily improved. By discharge, his renal function had stabilized, and inflammatory markers had decreased. He was hemodynamically stable on low-dose vasopressin and was transferred to a rehabilitation facility for continued physical therapy and nutritional care.

## 4. Conclusion

This case highlights the importance of early recognition and multidisciplinary management of dental aspiration. A high index of suspicion is crucial in cases of unexplained respiratory deterioration in ICU patients. Flexible bronchoscopy remains the cornerstone for diagnosis and treatment, especially in critically ill individuals. Moreover, reporting rare events such as dental aspiration is essential to enhance awareness among clinicians, refine diagnostic approaches, and inform tailored interventions. Multidisciplinary collaboration, including pulmonology, thoracic surgery, and critical care teams, plays an essential role in achieving a favorable outcome in such critically ill patients.

## Author contributions

**Investigation:** Hamza A. Abdul-Hafez, Mahdi Kittaneh, Abdulkarim Adas.

**Writing – original draft:** Hamza A. Abdul-Hafez, Enas Samara, Lana Saed, Mahdi Kittaneh, Abdulkarim Adas.

**Writing – review & editing:** Hamza A. Abdul-Hafez, Enas Samara, Lana Saed, Mahdi Kittaneh, Abdulkarim Adas.
